# Engineered Three-Dimensional Scaffolds Modulating Fate of Breast Cancer Cells Using Stiffness and Morphology Related Cell Adhesion

**DOI:** 10.1109/OJEMB.2020.2965084

**Published:** 2020-02-14

**Authors:** Samerender N. Hanumantharao, Carolynn A. Que, Brennan J. Vogl, Smitha Rao

**Affiliations:** Michigan Technological University 3968 Houghton MI 49931 USA; Department of Biomedical EngineeringMichigan Technological University 3968 Houghton MI 49931 USA

**Keywords:** 3D scaffold, breast cancer, cell adhesion, microenvironment, durotaxis, topotaxis

## Abstract

*Goal:* Artificially engineering the tumor microenvironment *in vitro* as a vital tool for understanding the mechanism of tumor progression. In this study, we developed three-dimensional cell scaffold systems with different topographical features and mechanical properties but similar surface chemistry. The cell behavior was modulated by the topography and mechanical properties of the scaffold. *Methods:* Adenocarcinoma (MCF7), triple-negative (MDA-MB-231) and premalignant (MCF10AneoT) breast cancer cells were seeded on the scaffold systems. The cell viability, cell-cell interaction and cell-matrix interactions were analyzed. The preferential growth and alignment of specific population of cells were demonstrated. *Results:* Among the different scaffolds, triple-negative breast cancer cells preferred honeycomb scaffolds while adenocarcinoma cells favored mesh scaffolds and premalignant cells preferred the aligned scaffolds. *Conclusions:* The 3D model system developed here can be used to support growth of only specific cell populations or for the growth of tumors. This model can be used for understanding the topographical and mechanical features affecting tumorigenesis, cancer cell growth and migration behavior of malignant and metastatic cancer cells.

## I. Introduction

The cancer microenvironment is a complex system consisting of extracellular matrix, stromal cells, adipocytes, fluids and vasculature [1]. This system is dynamically remodeled during tumorigenesis leading to a constantly evolving temporal and spatial 3D structures with distinct physical and pathophysiological alterations conducive to tumors [2]. The conventional 2D cell culture systems do not recapitulate the 3D tumor microenvironment. The cells grow in monolayers, lose polarity, and have an altered shape leading to changes in gene expression and splicing [3]–[7]. It fails to recreate the complex 3D intercellular signaling cascades and cell-matrix interactions, hypoxic conditions characteristic of tumor microenvironment, and communication between cells in different niches [8]–[10]. Three-dimensional systems such as tumor cell spheroids lack repeatability and are difficult to handle for animal studies [11]. The scaffolds provide a stable 3D environment for the cells to adhere, migrate, proliferate and differentiate [12]. They closely mimic the microenvironment with hypoxia-like conditions and cellular niches. Various materials, both natural (e.g., Engelbreth-Holm-Swam extract, collagen) and synthetic (polycaprolactone, poly(lactic-co-glycolic acid) have been used to fabricate scaffolds [13]–[15]. Recently, the adverse impact of using biological materials on immune cell recruitment was reported by Wolf and colleagues [16]. Synthetic polymer scaffolds have an advantage of being readily available and their production can be upscaled industrially [17]. Current 3D scaffold systems have limitations in design and connecting *in vivo* and *in vitro* conditions due to reductionist approaches. The synthetic material systems provide key information regarding cell migration and signaling cascades, but fail to consider the durotaxic and topotaxicmechanical properties and topographical cues, including roughness, curvature, porosity and fibrosity of the tumor microenvironment [18]–[22].

**Fig. 1.**
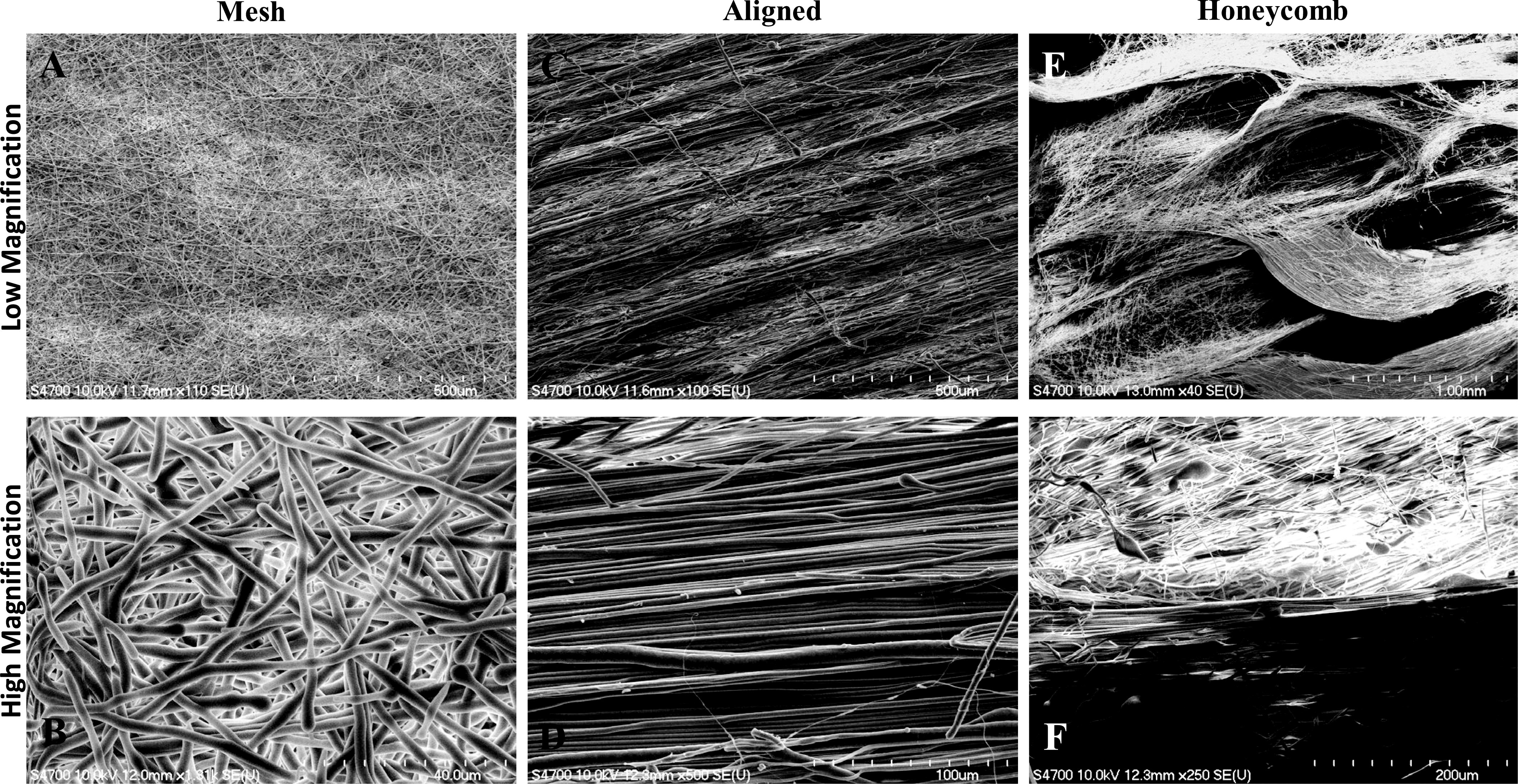
Field emission scanning electron microscopic (FESEM) images of the PCL scaffolds exhibiting different morphologies. The low magnification images (A, C, E) are present on the top while the high magnification images (B, D, F) are present in the bottom.

In this study, we engineered 3D scaffolds composed of well-defined morphologies and mechanical properties from polycaprolactone (PCL) using electrospinning [23], [24]. PCL is a synthetic, biodegradable, aliphatic polyester with slow and controllable degradation rates, and tunable mechanical properties [25]. Scaffolds with mesh, aligned and honeycomb morphologies (Fig. 1) were fabricated by manipulating the parameters used during electrospinning. Scaffolds with mesh morphology were designed to mimic the fibrous structure naturally present in the extracellular matrix of the breast tissue (ECM). The aligned morphology (naturally present in connective tissue) in scaffolds has previously been demonstrated to provide cues for durotaxis leading to differentiation, alignment of cells and as a predictor for breast cancer survival [26]–[30]. The porous honeycomb structure (naturally present in bones and alveolar tissue) mimicking the complex architecture present in tissues has been previously explored for tissue engineering [31]–[33], fibroblast growth and to inhibit the growth of cancer cells [34], [35]. Here we further investigate and delineate the effect of scaffold morphology on cancer cells. Breast cancer cell lines representing ductal adenocarcinoma (MCF-7), triple-negative metastatic (MDA-MB-231) and pre-malignant cancer (MCF10AneoT) were used to investigate the role of mechanical properties and topography on cancer cell adhesion and proliferation, and provide insights into the preferential behavior of cancer cells [36]. The scaffolds replicating different morphologies naturally present in the body helps in mimicking the conditions in vitro and explore the potential of topotactic and durotactic gradients of the extracellular matrix.

## II. Materials and Methods

### A. Fabrication of Scaffolds

All the materials were used as procured unless specified otherwise. Polycaprolactone (PCL): (Mw∼70,000 GPC; Scientific Polymer Products, USA) was used to obtain a sol-gel consisting of 20% PCL in chloroform (Sigma Aldrich, USA) for electrospinning. The voltage, rotational speed of the rotating collector and the polymer feed rate were varied as indicated in Table I (EM-DIG and EM-RTC; IME Technologies, Netherlands). Humidity, temperature, polymer fluid volume and tip-collector distance were constant.

**TABLE I:** Parameters Used for Electrospinning Scaffolds

Type of Scaffold	Voltage (kV)	Rotational Speed (RPM)	Polymer Feed Rate (µL/min)
Mesh	11	150	4
Aligned	11	275	4
Honeycomb	10	300	3
			

### B. Characterization of Scaffolds

The scaffolds were prepared by sputter coating with a 5 nm thick coating of Au/Pd for field emission scanning electron microscope (FESEM; Hitachi S-4700 FE-SEM). Fiji [37] was used for image analysis. The surface chemistry of the scaffolds was characterized using Attenuated Fourier Transform Infrared Spectroscopy (ATF-FTIR, Thermo Scientific, Nicolet iS50) with a deuterated triglycine sulfate detector element.  The measurements range of 400–4000 cm^−1^ at a resolution of 4 cm^−1^ with 256 scans was used. The mechanical properties of the scaffolds were determined by using a dynamic mechanical analyzer (TA Instruments, DMA Q800) under uniaxial strain ramp at isothermal conditions (37 °C). The Young's modulus was determined from the linear region of the stress-strain plot, uniaxial stiffness was determined from the force-displacement curve. The modulus of toughness and modulus of resilience were calculated from the area under the curve and area under the linear region of the stress-strain curve respectively.

### C. Cell Culture, Seeding, Viability and Immunochemistry

Breast ductal adenocarcinoma cancer cells (MCF7/ATCC HTB-22) and triple-negative malignant basal breast cancer cells (MDA-MB-231/ATCC HTB-26) were procured from American Type Cell Culture (ATCC). The premalignant cancer cell line, MCF10AneoT, was acquired from the Animal Model and Therapeutics Evaluation Core (AMTEC), Barbara Ann Karmanos Cancer Institute, Wayne State University. All cells were maintained under standard culture conditions and seeded on scaffolds as previously reported [33]. The scaffolds of 0.25 cm^2^ and 0.5 cm^2^ area (1500 cells) were used for cell viability and immunocytochemistry respectively following sterilization in ethanol and irradiation in UV. The cell viability (n = 9) was analyzed using CellTiter-Blue Cell Viability Assay (Promega, Madison, WI). Cells on tissue culture treated plates in similar conditions served as positive controls (n = 3). The fluorescence intensity was measured after 4 hours (Beckman Coulter DTX 880 Multimode Detector, ex/em 560 nm/590 nm). Fixed samples were permeabilized with Triton-X 100 and stained with Alexa Fluor 594 Phalloidin (Invitrogen, USA) and DAPI (4’,6-diamidino-2-phenylindole) (Life Technologies, USA) for visualizing the cytoskeletal F-Actin and A-T regions of the nucleus, respectively, according to the manufacturer's protocols.

### D. Statistical Analysis

Mechanical characterization of the scaffold was represented as mean ± SD (standard deviation). For cell viability, descriptive statistics was represented as mean ± SEM (standard error of mean). OriginPro 2018b and IBM SPSS statistics V25 was used for statistical evaluation of cell proliferation. One-way ANOVA followed by post-hoc Tukey's HSD test was used to calculate significance (p < 0.05) between days and difference between cell lines for each scaffold morphology.

## III. Results

### A. Topographical Characterization

**Fig. 2.**
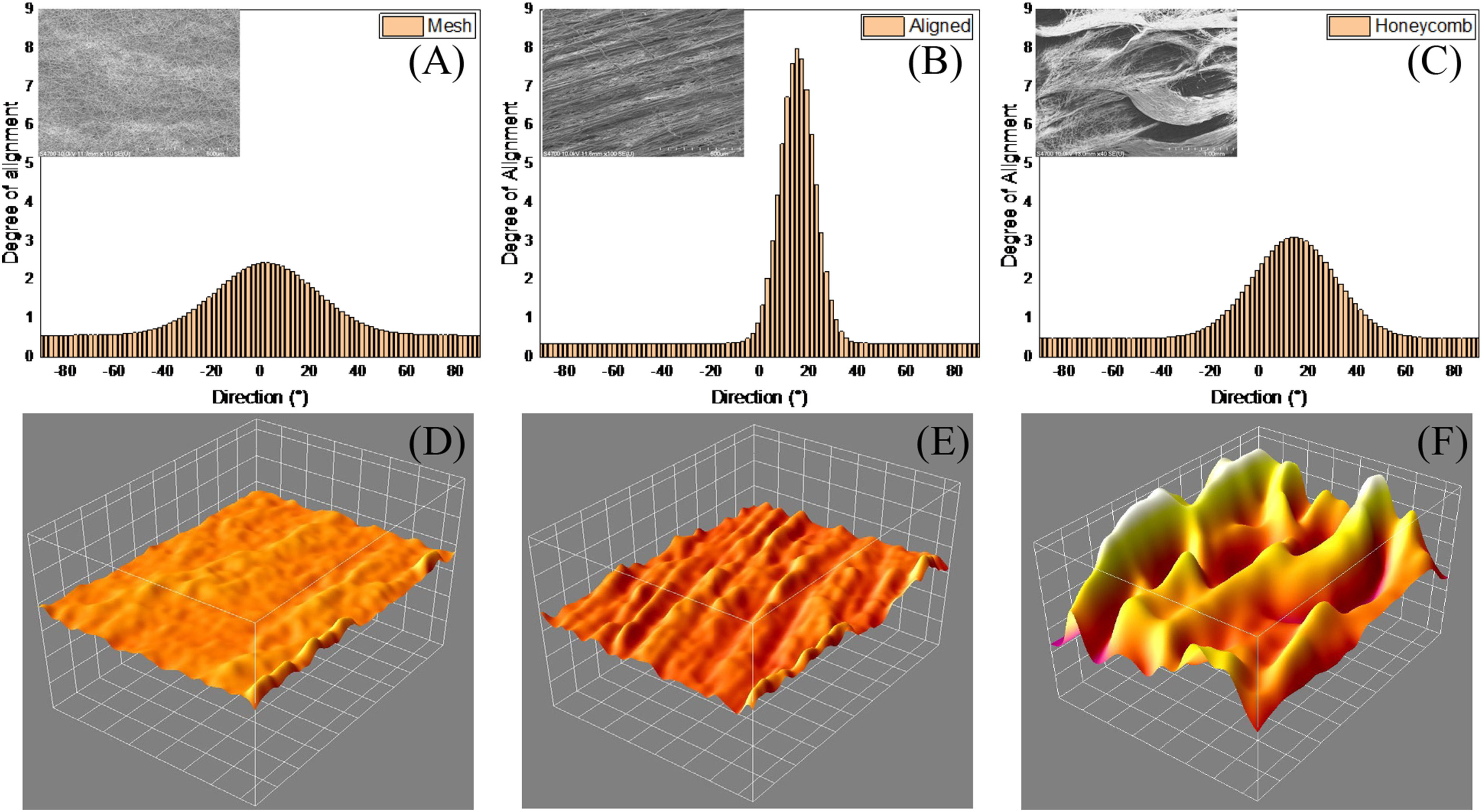
The degree of alignment and 3D topography scan of different morphologies of the scaffolds was characterized using the directionality plugin in ImageJ from the FESEM images used in Figure 1 as seen in the inlay (n = 5). (A) The mesh fibers had a high dispersion of fibers with low degree of alignment. (B) The aligned fibers had a high concentration of fibers in a narrow angle range with little deviation in other directions. (C) The honeycomb scaffolds had a broader range of deviation compared to the aligned scaffold but a much narrower distribution than the mesh scaffolds. (D) The fibers in mesh had little depth profile. (E) The fibers in aligned morphology had aligned fibers forming a pattern resembling grids (F) The fibers in the honeycomb morphology had well defined porous structure forming distinct regions.

From the FESEM images (Fig. 1), the formation of three distinct topographies can be inferred. The mesh scaffolds (Fig. 1(A)) have randomly oriented fibers, densely packed forming a 3D structure. The change in contrast (Fig. 1(B)) of the mesh like network indicates different layers. The aligned scaffolds had fibers tightly packed along an identical orientation. The scaffolds exhibited alternating regions of larger and smaller diameter fibers (Fig. 1(C)). A high concentration of overlapping aligned fibers in a tight network is visible in the high magnification (Fig. 1(D)). A low magnification image of the honeycomb scaffolds (Fig. 1(E)) composed of interlocking fibers in a specific pattern forming asymmetrical elongated honeycomb like structures. The structures had a long-range order and high aspect ratio. Densely packed fibers along the walls and aligned fibers at the bottom is visible in the high magnification image (Fig. 1(F)) of the boundary of the pores. The degree of alignment was the lowest in the mesh scaffolds (Fig. 2(A)) and highest in aligned scaffolds (Fig. 2(B)). The honeycomb morphology had a degree of alignment spread over a broad range of angles and a higher alignment of fibers than mesh but less than the aligned morphology (Fig. 2(C)). The fibers in mesh morphology had little depth with uniform topography (Fig. 2(D)), while the aligned morphology had fibers with a pattern resembling grids forming grooves (Fig. 2(E)). The porous structures were well defined and present throughout the honeycomb morphology forming distinct regions (Fig. 2(F)).

### B. Surface Chemistry

**Fig. 3.**
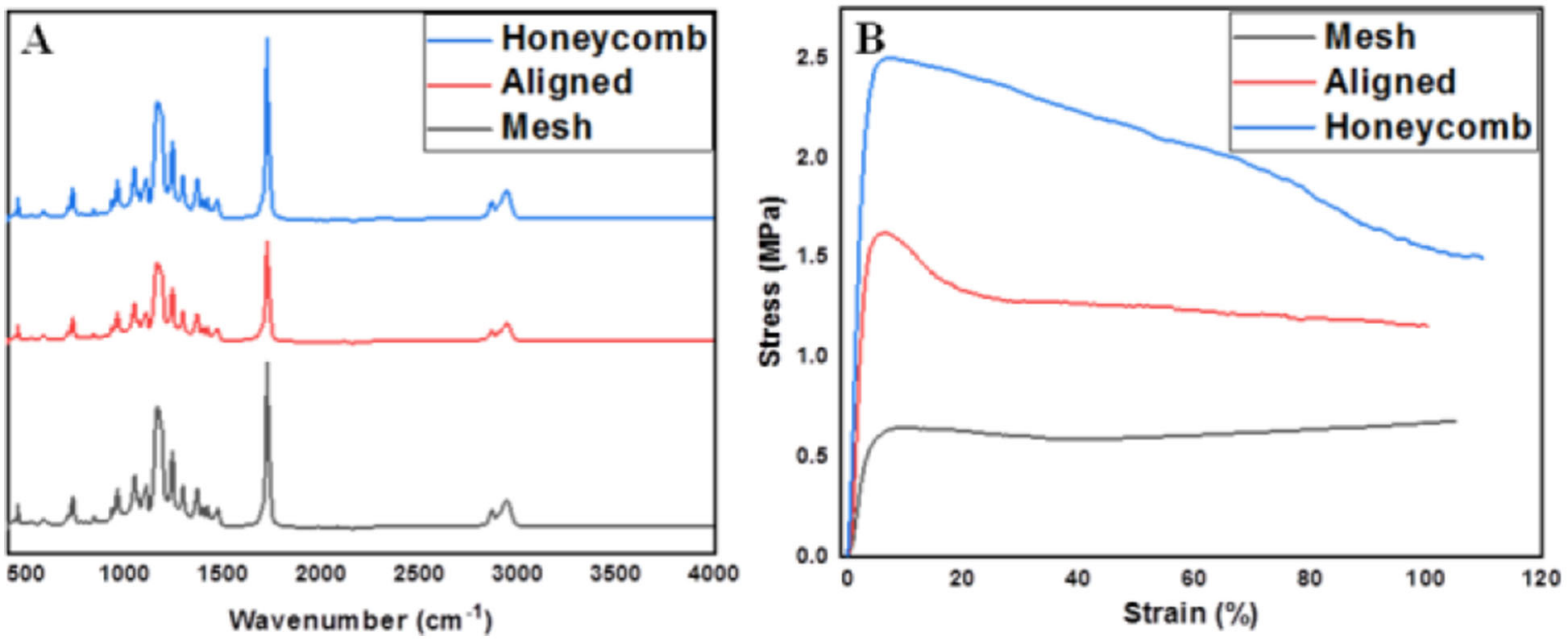
The surface and mechanical characterization of the PCL scaffolds of different morphologies was done. (A) Surface characterization was done using ATR-FTIR spectroscopy. The peaks distinctive to the molecular bond orientations present in PCL were identified. All the morphologies had similar surface chemistry. (B) The mechanical properties of the scaffolds was characterized using DMA at isothermal conditions (37 °C) and represented as stress-strain graph.

Surface characterization of the chemical bonds on the scaffolds was done using ATR-FTIR (Figs. 3(A) and S1). As the incident beam was focused on a larger surface area, it cannot be used to compare between the isotropic nature of the fibers in different scaffold morphologies. The stretching of the C-O and C-C bonds in the crystalline phase causes a peak at 1294 cm^−1^. The high electric field applied causes the PCL chains to orient along a direction accentuating the crystalling phase of PCL [38]. The peaks at 2942 cm^−1^ and 2865 cm^−1^ represent asymmetric and symmetric stretching of the CH_2_ group. The peak at 1723 cm^−1^ corresponds to C = O vibration of ester. The bands at 1239 cm^−1^ and 1165 cm^−1^ are associated with asymmetric and symmetric stretching of the ester COO group. The peak at 1365 cm^−1^ correspond to the CH_2_ band vibrations while the O–C vibrations and CH_2_ vibration occur at 961 cm^−1^ and 732 cm^−1^ respectively.

### C. Mechanical Properties

The mechanical characterization of the scaffolds (Table II) was done using DMA at isothermal conditions (Fig. 3(B)). The stress-strain behavior of the scaffolds was unique to each morphology. The honeycomb scaffold has the highest average ultimate strength and stiffness. The aligned scaffolds have mechanical properties comparable to the honeycomb scaffolds with respect to the Young's modulus and stiffness, however the modulus of toughness is lower than honeycomb scaffolds. The mesh scaffolds have relatively poor strength and toughness. All three morphologies have a non-significant difference in strain at failure.

**TABLE II:** Mechanical Properties of the Different Morphologies of the Scaffold

	Mesh	Aligned	Honeycomb
Young's Modulus (MPa)	0.155 ± 0.01	0.560 ± 0.12	0.569 ± 0.14
Modulus of Resilience	1.603 ± 0.64	2.945 ± 0.59	5.547 ± 0.04
Ultimate Tensile Strength (MPa)	0.818 ± 0.12	1.549 ± 0.07	2.341 ± 0.14
Strain at Failure (%)	104.512 ± 1.09	106.736 ± 5.99	107.512 ± 4.60
Modulus of Toughness	76.473 ± 10.63	136.885 ± 9.41	189.273 ± 28.40

### D. Immunocytochemistry

Breast cancer cell lines representing various stages of cancer progression (adenocarcinoma, premalignant, triple-negative/metastatic) were used to evaluate the behavior of cancer cells on different topographies and mechanical properties of the scaffold. The cells were stained and fixed on days 1, 2 and 3. Qualitative analysis of the behavior of the cells to changes in morphology of the scaffold was assessed by immunocytochemistry on fixed cells on days 1, 2, and 3 after seeding. High magnification images of some of the phenotypes used for characterizing the behavior of the scaffolds is shown in Figure S2.

#### 1) MCF7

**Fig. 4.**
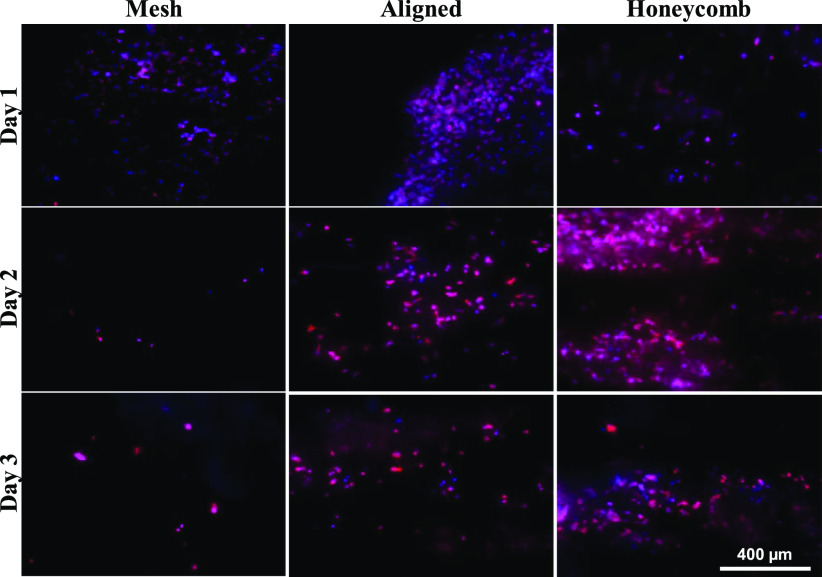
Fluorescent microscope images of Adenocarcinoma cells (MCF7) on different morphologies of the PCL scaffold on days 1, 2 and 3. The nuclei were stained with DAPI (blue) and the F-actin filaments were stained with Alexa Fluor 594 Phalloidin (red). The overlapping of the blue and red and dispersion by the fibers causes some of the cells to be seen pink in color. Images captured at 10X magnification.

From Figs. 4 and S3, the cells were clumped on all three morphologies on day 1 with extensive clumping in the aligned scaffolds. In the mesh morphology, on days 2 and 3, cells infiltrated the scaffold and were spread affecting imaging. In the aligned scaffold, on days 2 and 3 oriented along the direction of alignment of the fibers and were spread out with an elongated morphology. The honeycomb scaffold had a high concentration of cells in the pores and almost negligible number of cells on the boundary of the pores on day 2. On day 3, the cells infiltrated the layers of fibers and were present in between the fibers (blurred regions between the fibers). Based on the cellular distribution, morphology and orientation (day 3), it appears that the cells responded to the nanotopographical cues, distinctive of the morphologies of the scaffolds.

#### 2) MDA-MB-231

**Fig. 5.**
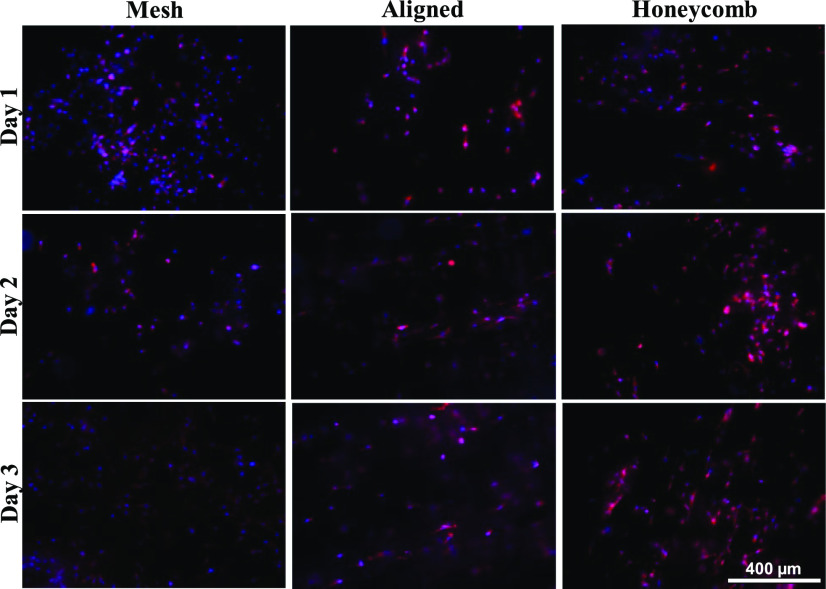
Fluorescent microscope images of triple negative breast cancer cells (MDA-MB-231) on different morphologies of the PCL scaffold on days 1, 2 and 3. The nuclei were stained with DAPI (blue) and the F-actin filaments were stained with Alexa Fluor 594 Phalloidin (red). The overlapping of the blue and red and dispersion by the fibers causes some of the cells to be seen pink in color. Images captured at 10X magnification.

From Figs. 5 and S4, the cells were distributed across the mesh scaffold without any orientation on all three days. In the aligned scaffold, the cells lacked alignment on day 1, but were spread out. Cell alignment and elongation was along the fiber alignment on day 2. On day 3, the cells infiltrated the layers (blurred background) on day 3. There was little cellular alignment in the honeycomb on day 1. However, this improved on day 2 and the cells infiltrated the scaffolds with preferential attachment to the walls rather than the underlying layers of the pores. Clumping was not observed in any of the morphologies.

#### 3) MCF10AneoT

**Fig. 6.**
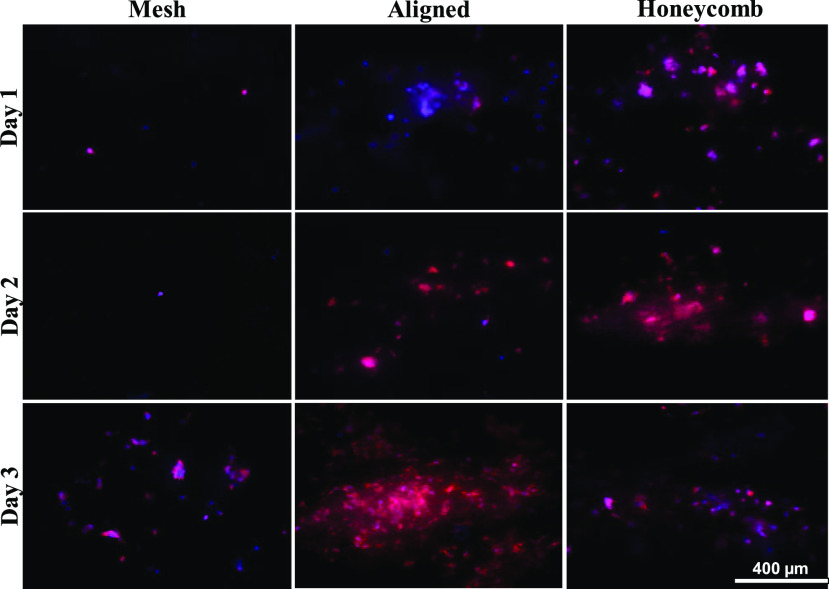
Fluorescent microscope images of premalignant breast cancer cells (MCF10AneoT) on different morphologies of the PCL scaffold on days 1, 2 and 3. The nuclei were stained with DAPI (blue) and the F-actin filaments were stained with Alexa Fluor 594 Phalloidin (red). The overlapping of the blue and red and dispersion by the fibers causes some of the cells to be seen pink in color. Images captured at 10X magnification.

From Figs. 6 and S5, the cells appear clumped in all the scaffold morphologies for all days with infiltration. In the mesh scaffold, the clumping was localized and lacked cellular orientation. In the aligned scaffolds, the cells were spread out with minimal cellular alignment on days 1 and 2 but, appear to align along the fibers on day 3. In the honeycomb scaffold, cell infiltration was observed after day 1, with increased infiltration on days 2 and 3. The cells on day 3 did not present the clumping observed on day 2.

### E. Cell Viability

**Fig. 7.**
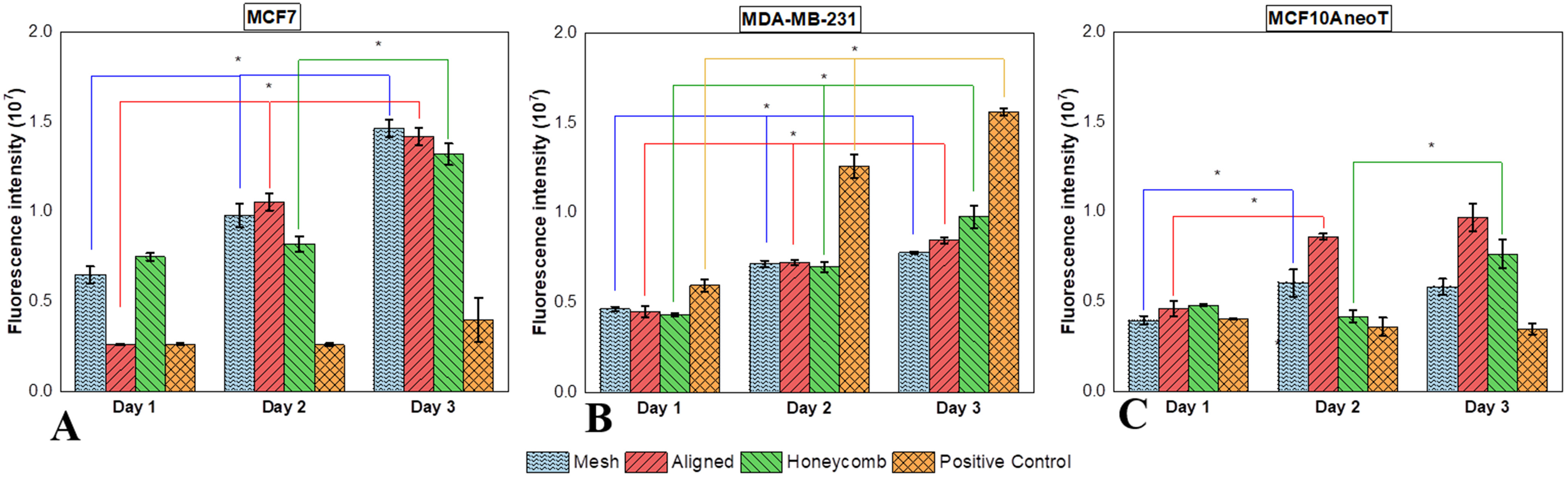
Quantification of cell viability on days 1, 2 and 3 on different morphologies and cell lines. (A) Cell viability of MCF7 (adenocarcinoma) cells on different morphologies of the scaffold. Cells seeded on mesh scaffold had a significant increase in cell number till day 3. (B) Cell viability of MDA-MB-231 (triple negative) cells on different morphologies of the scaffold. The cell number increased consistently on all scaffolds till day 3. (C) Cell viability of MCF10AneoT (premalignant) cells on different morphologies of the scaffold. The increase in cell viability was inconsistent across any morphology. Error bars represent the standard error of mean and statistical significance is indicated by p values (^*^p < 0.05).

The cell viability on scaffolds on days 1, 2 and 3 were analyzed by characterizing the reduction of resazurin to resorufin. MCF7 had a significant increase in cell number across all scaffold morphologies on all days (Fig. 7(A)). The number of viable cells was lowest in the positive control with non-significant increase for each day. While the MDA-MB-231 cells had a statistically significant increase in cell viability over all the days on all the morphologies (Fig. 7(B)), the positive control was the most favorable. The viability of MCF10AneoT cells (Fig. 7(C)) was not uniform. The increase in cell number was more pronounced from day 1 to day 2 in the mesh and aligned scaffolds and from day 2 to day 3 in honeycomb scaffolds with non-significant increase in the positive control. The increase in cell number for MDA-MB-231 and MCF7 was uniform in all scaffold morphologies.

## IV. Discussion

The parameters used for electrospinning were varied to form fibrous scaffolds with different morphologies from the same polymer blend eliminating variability introduced by using different materials or processing. The applied electric field determines the initial elastic stress and the bending instabilities in the jet [39] and to control the spatial deposition of fibers essential in creating the topographical features [40]. The rotational velocity of the collector helps in controlling the orientation, diameter and alignment [41]. However, the critical rotational velocity is determined by the applied average electric field. A lower rotational speed of the collector yields less alignment whereas higher rotational speed yields fibers with orientation perpendicular to the electric field vector. The average electric field was increased along with the rotational speed to fabricate novel three-dimensional honeycomb shaped scaffolds. The dense fiber network helps to mimic conditions of tumor-induced angiogenesis as reported by Bauer and colleagues [42], [43].

The mesh scaffolds behave like an elastomer owing to the low rotational velocity of the collector and voltage applied during electrospinning [44]. The aligned scaffolds have better orientation in the microcrystalline regions because of their morphology increasing the Young's modulus of the scaffold [45], [46]. The honeycomb structure by design has superior mechanical properties than other morphologies. This is due to loss in morphology with increasing strain and the fibers aligning in the direction of stress.

The cell viability, cell-cell interaction and cell-scaffold behavior were influenced by the topographical features and mechanical properties of the scaffolds. The MCF7 cells proliferated well in all the scaffolds tested without any preference to a particular topographical feature. This agrees with the findings by Chaudhuri *et al.* on the inhibition of Rho-ROCK-Myosin signaling in malignant cells leading to proliferation of adenocarcinoma irrespective of the topography [47]. However, the cells preferred elastomeric scaffolds with low Young's modulus and stiffness (mesh) over compared to the honeycomb and the positive control. This agrees well with the experimental investigation on mechanical properties of the MCF7 cells through atomic force microscopy measurements by Li *et al.* [48] and durotaxis studies by Cavo *et al.* [49]. The triple-negative cells on the other hand thrived in scaffolds with high matrix stiffness, as expected due to regulation of the YAP (Yes-associated protein)/TAZ (transcriptional coactivator with PDZ-binding motif) and subsequent activation of the Hippo cascade [50]. The stiffness of the positive control (tissue culture plate ∼10GPa) plays a major role in the cellular viability and agrees well with the findings by Mah *et al.* [51]. On the honeycomb and aligned scaffolds, the cell alignment and infiltration were guided by the topography and mechanical properties of the scaffold, demonstrating extensive cellular infiltration and alignment. The stiffness of the scaffolds also positively enhances the migration potential of the metastatic cells as reported by Lin *et al.* [52] and can drive tumor progression through a TWIST1-G3BP2 mechanotransduction pathway [53]. The premalignant cells preferred the aligned scaffolds and infiltrated and aligned along the orientation of the fibers. The topographical cues provided by the aligned scaffolds helps in cell spreading and can also impact tumor progression and metastasis [54]. However, there was a certain amount of clumping in the cells in all the morphologies. This clumping is directly correlated to the metastatic potential of the cells where, the clumped cells form protrusions followed by invasion [55], [56]. The increased viability and spreading of the premalignant cells in stiffer scaffolds are consistent with the observations made by Rubashkin *et al.* [57]. Thus, it can be concluded that the cells respond to changes in scaffold topography and mechanical properties based on the stage of cancer, effectively providing a suitable *in vitro* model. The scaffold provides an ideal platform for studying breast cancer metastasis or for localized therapy to inhibit the growth of metastatic cells. The efficient scaffold design also allows the system to be easily adopted for the study and treatment of other cancers through the respective durotaxic and topotaxic gradients [58].

## V. Conclusions

Three-dimensional scaffolds with different topographies and mechanical properties were fabricated using electrospinning from polycaprolactone (PCL) with similar surface chemistry. Adenocarcinoma, triple-negative and premalignant breast cancer cells were seeded on scaffolds with different morphologies and characterized. Cell-cell and cell-scaffold interaction was qualitatively analyzed and the cell viability across all the days were quantitatively assessed. The triple-negative cells preferred honeycomb scaffolds with higher stiffness and strength, while adenocarcinoma cells proliferated favorably on mesh scaffolds with low elastic modulus and premalignant cells favored aligned scaffolds with high stiffness and greater contact guidance. The current study can be used to design scaffolds which can mimic the tumor microenvironment and for selectively modeling cancer cell population in an *in vitro* 3D system.

## Supplementary Materials

The online-only supplementary material has additional information regarding the surface characterization of the PCL scaffolds using FTIR, high magnification images of the phenotype of cells discussed in the paper and fluorscent-phase overlay images of the different cell lines on different morphologies of the PCL scaffolds.


