# Supplementary material for: Engineered Three-Dimensional Scaffolds Modulating Fate of Breast Cancer Cells Using Stiffness and Morphology Related Cell Adhesion
Source: IEEE Open J Eng Med Biol. 2020 Feb 14;1:41–8. doi: 10.1109/OJEMB.2020.2965084 (PMC8979620; doi:10.1109/OJEMB.2020.2965084)
Supplement: Supplementary file 1 [file supp1-2965084.pdf]

Supplementary Materials

Engineered three-dimensional scaffolds modulating fate of breast cancer cells using stiffness and morphology related cell adhesion

Samerender N. Hanumantharao, Carolynn A. Que, Brennan J. Vogl, Smitha Rao*, *Senior Member, IEEE*

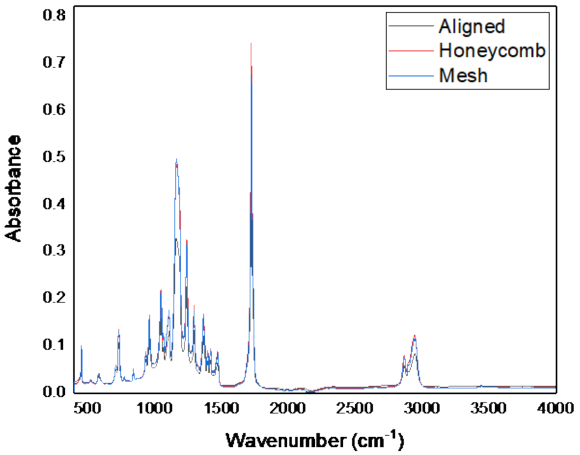

Fig. S1. The surface characterization of the PCL scaffolds of different morphologies was done. The spectrum is overlapped to show the similarity in absorbances of characteristics bonds of PCL from different morphologies of scaffolds.

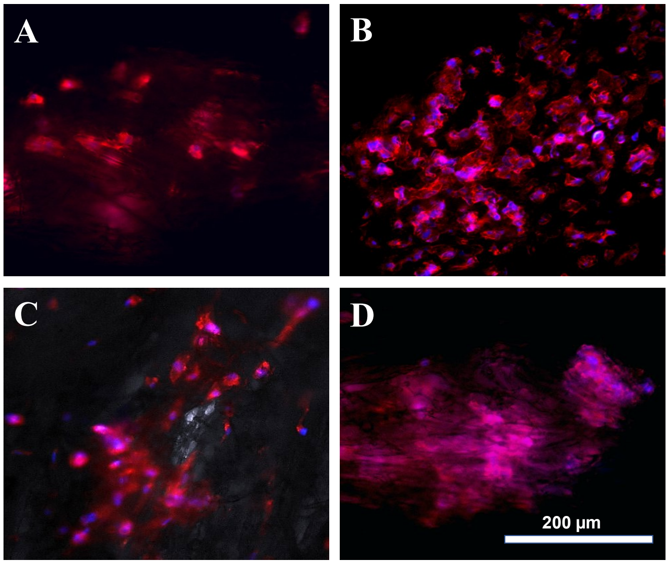

Fig. S2. Phenotypes of the cells on scaffolds used to define cell behavior on the different morphologies of the scaffold. The phase images show the fibers and the overlaid fluorescent images show the alignment of nucleus against the orientation of the fibers. A) Aligned and elongated cells infiltrating into the scaffold. B) Clumped cells on scaffolds C) Cells aligning along the alignment of the scaffold D) The cells infiltrating through the different layers of the scaffolds in clumps.

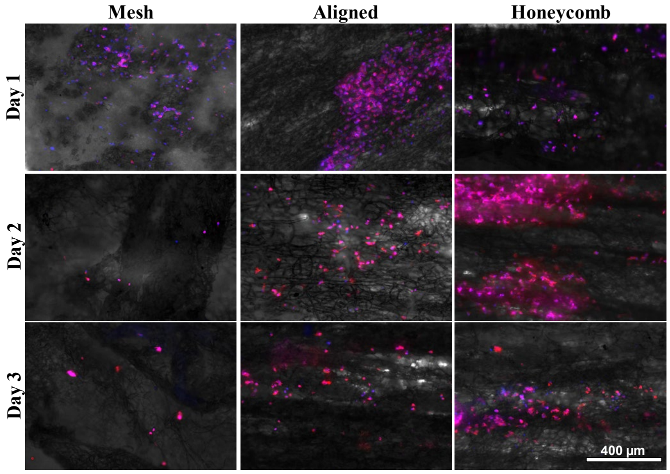

Fig. S3. Fluorescent microscope images of adenocarcinoma cells (MCF7) on different morphologies of the PCL scaffold on days 1, 2 and 3. The nuclei were stained with DAPI (blue) and the F-actin filaments were stained with Alexa Fluor® 594 Phalloidin (red). The images were overlaid with the phase contrast image to provide additional information about the scaffold morphology. The overlapping of the blue and red and dispersion by the fibers causes some of the cells to be seen pink in color. Images captured at 10X magnification.

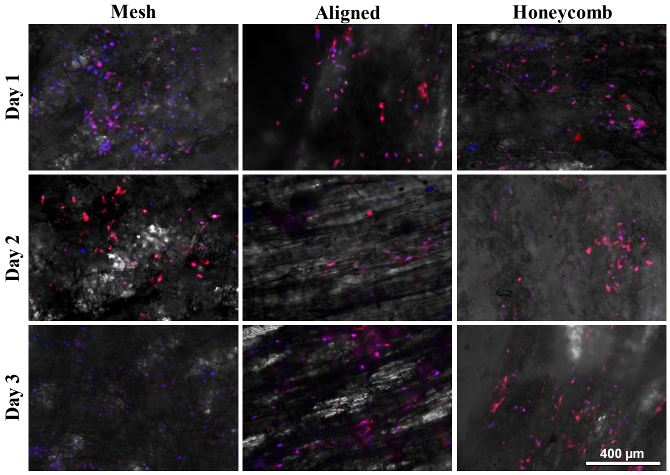

Fig. S4. Fluorescent microscope images of triple negative breast cancer cells (MDA-MB-231) on different morphologies of the PCL scaffold on days 1, 2 and 3. The nuclei were stained with DAPI (blue) and the F-actin filaments were stained with Alexa Fluor® 594 Phalloidin (red). The images were overlaid with the phase contrast image to provide additional information about the scaffold morphology. The overlapping of the blue and red and dispersion by the fibers causes some of the cells to be seen pink in color. Images captured at 10X magnification.

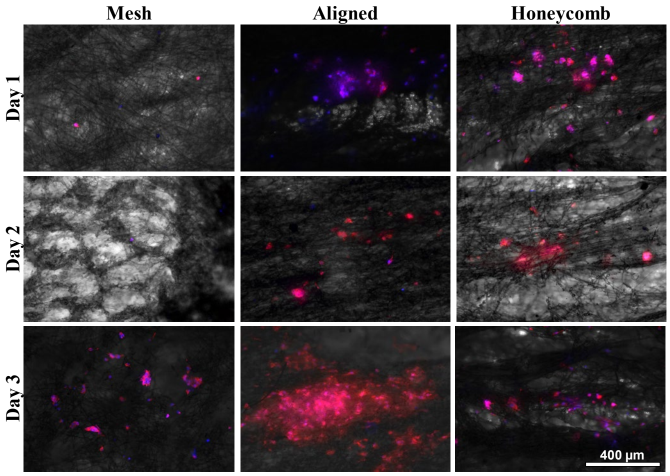

Fig. S5. Fluorescent microscope images of premalignant breast cancer cells (MCF10AneoT) on different morphologies of the PCL scaffold on days 1, 2 and 3. The nuclei were stained with DAPI (blue) and the F-actin filaments were stained with Alexa Fluor® 594 Phalloidin (red). The images were overlaid with the phase contrast image to provide additional information about the scaffold morphology. The overlapping of the blue and red and dispersion by the fibers causes some of the cells to be seen pink in color. Images captured at 10X magnification.
